# The Role of Uremic Toxins in Comorbidities of Chronic Kidney Disease

**DOI:** 10.3390/toxins18090387

**Published:** 2026-09-07

**Authors:** Suguru Yamamoto

**Affiliations:** Division of Clinical Nephrology and Rheumatology, Kidney Research Center, Niigata University Graduate School of Medicine, Dentistry and Health Sciences, Niigata 951-8510, Japan; yamamots@med.niigata-u.ac.jp

The number of patients with chronic kidney disease (CKD) has increased worldwide. Although treatments to slow the progression of CKD and address CKD-related systemic disorders have advanced, the mortality risk for CKD patients, particularly those on dialysis, remains high. Additionally, both physical activity and quality of life are impaired in CKD patients, especially those undergoing dialysis treatment. Given the association between longer dialysis duration and the onset of frailty, it is crucial to identify and address unknown and unresolved CKD-related factors and develop new treatment strategies.

This Special Issue, titled “The Role of Uremic Toxins in Comorbidities of Chronic Kidney Disease,” aims to explore the roles of uremic toxins in CKD-related systemic disorders and their treatment. The collection brings together 11 papers—eight original research articles and three reviews—that examine uremic toxicity from complementary basic, clinical, nutritional, vascular, and therapeutic perspectives. Collectively, these contributions reinforce the view that retained solutes are not merely markers of reduced kidney function, but active participants in the systemic complications of chronic kidney disease (CKD).

Obrero et al. showed that probiotic supplementation may modulate gut-derived uremic toxin production, inflammation, mineral metabolism, and vascular calcification in experimental and clinical CKD models [1]. Malaweera et al. clarified the associations among protein-bound uremic toxins, dietary fibre intake, and patient-centred well-being in individuals receiving kidney replacement therapy [2]. Hardjo et al. mapped the development of uremic toxin research and identified major trends, key molecules, and unresolved topics through a comprehensive bibliometric analysis [3].

Improved cardiovascular risk markers were reported in patients undergoing hemodialysis following apitherapy with royal jelly and green propolis EPP-AF^®^ (*Toxins*, 2025, 17(8), 369). Serum indoxyl sulfate was identified as a potential biomarker of peripheral arterial stiffness in patients with non-dialysis CKD stages 3 to 5 (*Toxins*, 2025, 17(6), 283). Myostatin was shown to exacerbate endothelial dysfunction induced by indoxyl sulfate and to be associated with hemodialysis arteriovenous access complications (*Toxins*, 2025, 17(4), 159). The role of indoxyl sulfate in exacerbating colorectal cancer during CKD progression through the Akt/β-catenin/c-Myc and AhR/c-Myc pathways was examined in HCT-116 cells (*Toxins*, 2025, 17(1), 17). A meta-analysis showed that lowering serum indoxyl sulfate with AST-120 improved renal outcomes and the lipid profile in diabetic and nondiabetic animal models of CKD (*Toxins*, 2024, 16(12), 544). Molecular pathways and integrated phenotypes underlying uremic toxin-driven vascular calcification in CKD were reviewed (*Toxins*, 2026, 18(2), 112). The pathological role of LDL in membranous nephropathy and diabetic nephropathy, together with the protective efficacy of LDL apheresis, was reviewed (*Toxins*, 2026, 18(1), 29). The current understanding of calcimimetics and vascular calcification was summarized (*Toxins*, 2025, 17(6), 297).

Several themes emerge across these papers. First, protein-bound uremic toxins remain a central molecule linking CKD to vascular dysfunction, dialysis-access complications, and potentially carcinogenic signalling. Second, the gut–kidney axis and dietary factors offer practical targets for reducing toxin generation and improving outcomes that matter to patients. Third, vascular calcification and cardiovascular injury arise from interacting toxic, inflammatory, metabolic, and endocrine pathways rather than from a single abnormality. Finally, the collection underscores the need to connect mechanistic discoveries with clinically useful biomarkers and interventions.

Important questions nevertheless remain. Larger prospective studies are required to define clinically meaningful toxin thresholds, clarify causal relationships, and determine whether lowering specific toxins improves survival, cardiovascular events, physical function, and quality of life. Standardized analytical methods and closer integration of multi-omics, nutrition, dialysis technology, and patient-reported outcomes will be essential for translating uremic toxin research into routine care.

I hope that this Special Issue will stimulate continued collaboration and accelerate progress toward reducing the burden of uremic toxicity and its comorbidities in people living with CKD.
